# Supplementary material for: Human BRCA pathogenic variants were originated during recent human history
Source: Life Sci Alliance. 2022 Feb 14;5(5):e202101263. doi: 10.26508/lsa.202101263 (PMC8860097; doi:10.26508/lsa.202101263)
Supplement: Supplementary file 6 [file LSA-2021-01263_TableS6.docx]

**References for ancient human genomes and founder mutations**

1. Agranat-Tamir, L. et al. The Genomic History of the Bronze Age Southern Levant. Cell 181, 1146-1157 e1111, doi:10.1016/j.cell.2020.04.024 (2020).
2. Damgaard, P. B. et al. 137 ancient human genomes from across the Eurasian steppes. Nature 557, 369-374, doi:10.1038/s41586-018-0094-2 (2018).
3. Fernandes, D. M. et al. The spread of steppe and Iranian-related ancestry in the islands of the western Mediterranean. Nat Ecol Evol 4, 334-345, doi:10.1038/s41559-020-1102-0 (2020).
4. Fernandes, D. M. et al. A genetic history of the pre-contact Caribbean. Nature 590, 103-110, doi:10.1038/s41586-020-03053-2 (2021).
5. Flegontov, P. et al. Palaeo-Eskimo genetic ancestry and the peopling of Chukotka and North America. Nature 570, 236-240, doi:10.1038/s41586-019-1251-y (2019).
6. Fleskes, R. E. et al. Ancient DNA and bioarchaeological perspectives on European and African diversity and relationships on the colonial Delaware frontier. Am J Phys Anthropol 170, 232-245, doi:10.1002/ajpa.23887 (2019).
7. Fu, Q. et al. The genetic history of Ice Age Europe. Nature 534, 200-205, doi:10.1038/nature17993 (2016).
8. Haak, W. et al. Massive migration from the steppe was a source for Indo-European languages in Europe. Nature 522, 207-211, doi:10.1038/nature14317 (2015).
9. Harney, E. et al. A minimally destructive protocol for DNA extraction from ancient teeth. Genome Res 31, 472-483, doi:10.1101/gr.267534.120 (2021).
10. Harney, E. et al. Ancient DNA from Chalcolithic Israel reveals the role of population mixture in cultural transformation. Nat Commun 9, 3336, doi:10.1038/s41467-018-05649-9 (2018).
11. Harney, E. et al. Ancient DNA from the skeletons of Roopkund Lake reveals Mediterranean migrants in India. Nat Commun 10, 3670, doi:10.1038/s41467-019-11357-9 (2019).
12. Lazaridis, I. et al. Genetic origins of the Minoans and Mycenaeans. Nature 548, 214-218, doi:10.1038/nature23310 (2017).
13. Lazaridis, I. et al. Genomic insights into the origin of farming in the ancient Near East. Nature 536, 419-424, doi:10.1038/nature19310 (2016).
14. Lazaridis, I. et al. Ancient human genomes suggest three ancestral populations for present-day Europeans. Nature 513, 409-413, doi:10.1038/nature13673 (2014).
15. Lipson, M. et al. Ancient genomes document multiple waves of migration in Southeast Asian prehistory. Science 361, 92-95, doi:10.1126/science.aat3188 (2018).
16. Lipson, M. et al. Ancient West African foragers in the context of African population history. Nature 577, 665-670, doi:10.1038/s41586-020-1929-1 (2020).
17. Lipson, M. et al. Population Turnover in Remote Oceania Shortly after Initial Settlement. Curr Biol 28, 1157-1165 e1157, doi:10.1016/j.cub.2018.02.051 (2018).
18. Lipson, M. et al. Three Phases of Ancient Migration Shaped the Ancestry of Human Populations in Vanuatu. Curr Biol 30, 4846-4856 e4846, doi:10.1016/j.cub.2020.09.035 (2020).
19. Lipson, M. et al. Parallel palaeogenomic transects reveal complex genetic history of early European farmers. Nature 551, 368-372, doi:10.1038/nature24476 (2017).
20. Mathieson, I. et al. The genomic history of southeastern Europe. Nature 555, 197-203, doi:10.1038/nature25778 (2018).
21. Mathieson, I. et al. Genome-wide patterns of selection in 230 ancient Eurasians. Nature 528, 499-503, doi:10.1038/nature16152 (2015).
22. Nakatsuka, N. et al. A Paleogenomic Reconstruction of the Deep Population History of the Andes. Cell 181, 1131-1145 e1121, doi:10.1016/j.cell.2020.04.015 (2020).
23. Nakatsuka, N. et al. Ancient genomes in South Patagonia reveal population movements associated with technological shifts and geography. Nat Commun 11, 3868, doi:10.1038/s41467-020-17656-w (2020).
24. Narasimhan, V. M. et al. The formation of human populations in South and Central Asia. Science 365, doi:10.1126/science.aat7487 (2019).
25. Nikitin, A. G. et al. Interactions between earliest Linearbandkeramik farmers and central European hunter gatherers at the dawn of European Neolithization. Sci Rep 9, 19544, doi:10.1038/s41598-019-56029-2 (2019).
26. Olalde, I. et al. The Beaker phenomenon and the genomic transformation of northwest Europe. Nature 555, 190-196, doi:10.1038/nature25738 (2018).
27. Olalde, I. et al. The genomic history of the Iberian Peninsula over the past 8000 years. Science 363, 1230-1234, doi:10.1126/science.aav4040 (2019).
28. Prendergast, M. E. et al. Ancient DNA reveals a multistep spread of the first herders into sub-Saharan Africa. Science 365, doi:10.1126/science.aaw6275 (2019).
29. Shinde, V. et al. An Ancient Harappan Genome Lacks Ancestry from Steppe Pastoralists or Iranian Farmers. Cell 179, 729-735 e710, doi:10.1016/j.cell.2019.08.048 (2019).
30. Sikora, M. et al. The population history of northeastern Siberia since the Pleistocene. Nature 570, 182-188, doi:10.1038/s41586-019-1279-z (2019).
31. Sirak, K. et al. Human auditory ossicles as an alternative optimal source of ancient DNA. Genome Res 30, 427-436, doi:10.1101/gr.260141.119 (2020).
32. Skoglund, P. et al. Genomic insights into the peopling of the Southwest Pacific. Nature 538, 510-513, doi:10.1038/nature19844 (2016).
33. Unterlander, M. et al. Ancestry and demography and descendants of Iron Age nomads of the Eurasian Steppe. Nat Commun 8, 14615, doi:10.1038/ncomms14615 (2017).
34. Wang, T. et al. Human population history at the crossroads of East and Southeast Asia since 11,000 years ago. Cell 184, 3829-3841 e3821, doi:10.1016/j.cell.2021.05.018 (2021).
35. Yang, M. A. et al. Ancient DNA indicates human population shifts and admixture in northern and southern China. Science 369, 282-288, doi:10.1126/science.aba0909 (2020).
36. Skoglund, P. et al. Reconstructing Prehistoric African Population Structure. Cell 171, 59-71 e21, doi:10.1016/j.cell.2017.08.049 (2017).
37. Schlebusch, C. M. et al. Southern African ancient genomes estimate modern human divergence to 350,000 to 260,000 years ago. Science 358, 652-655, doi:10.1126/science.aao6266 (2017).
38. Seguin-Orlando, A. et al. Paleogenomics. Genomic structure in Europeans dating back at least 36,200 years. Science 346, 1113-1118, doi:10.1126/science.aaa0114 (2014).
39. Tuazon, A. M. A. et al. Haplotype analysis of the internationally distributed BRCA1 c.3331_3334delCAAG founder mutation reveals a common ancestral origin in Iberia. Breast Cancer Res 22, 108, doi:10.1186/s13058-020-01341-3 (2020).
40. Hamel, N. et al. On the origin and diffusion of BRCA1 c.5266dupC (5382insC) in European populations. Eur J Hum Genet 19, 300-306, doi:10.1038/ejhg.2010.203 (2011).
41. Cini, G. et al. Tracking of the origin of recurrent mutations of the BRCA1 and BRCA2 genes in the North-East of Italy and improved mutation analysis strategy. BMC Med Genet 17, 11, doi:10.1186/s12881-016-0274-6 (2016).
42. Laitman, Y. et al. Haplotype analysis of the 185delAG BRCA1 mutation in ethnically diverse populations. Eur J Hum Genet 21, 212-216, doi:10.1038/ejhg.2012.124 (2013).
43. Bergman, A. et al. The western Swedish BRCA1 founder mutation 3171ins5; a 3.7 cM conserved haplotype of today is a reminiscence of a 1500-year-old mutation. Eur J Hum Genet 9, 787-793, doi:10.1038/sj.ejhg.5200704 (2001).
44. Weitzel, J. N. et al. Prevalence and type of BRCA mutations in Hispanics undergoing genetic cancer risk assessment in the southwestern United States: a report from the Clinical Cancer Genetics Community Research Network. J Clin Oncol 31, 210-216, doi:10.1200/JCO.2011.41.0027 (2013).
45. Herzog, J. S. et al. Genetic epidemiology of BRCA1- and BRCA2-associated cancer across Latin America. NPJ Breast Cancer 7, 107, doi:10.1038/s41523-021-00317-6 (2021).
46. Quiles, F. et al. Identification of a founder BRCA1 mutation in the Moroccan population. Clin Genet 90, 361-365, doi:10.1111/cge.12747 (2016).
47. Marroni, F. et al. Reconstructing the genealogy of a BRCA1 founder mutation by phylogenetic analysis. Ann Hum Genet 72, 310-318, doi:10.1111/j.1469-1809.2007.00420.x (2008).
48. Neuhausen, S. L. et al. Haplotype and phenotype analysis of six recurrent BRCA1 mutations in 61 families: results of an international study. Am J Hum Genet 58, 271-280 (1996).
49. Dorum, A., Heimdal, K., Hovig, E., Inganas, M. & Moller, P. Penetrances of BRCA1 1675delA and 1135insA with respect to breast cancer and ovarian cancer. Am J Hum Genet 65, 671-679, doi:10.1086/302530 (1999).
50. Moller, P. et al. Genetic epidemiology of BRCA1 mutations in Norway. Eur J Cancer 37, 2428-2434, doi:10.1016/s0959-8049(01)00299-4 (2001).
51. Reeves, M. D. et al. BRCA1 mutations in South African breast and/or ovarian cancer families: evidence of a novel founder mutation in Afrikaner families. Int J Cancer 110, 677-682, doi:10.1002/ijc.20186 (2004).
52. Infante, M. et al. BRCA1 5272-1G>A and BRCA2 5374delTATG are founder mutations of high relevance for genetic counselling in breast/ovarian cancer families of Spanish origin. Clin Genet 77, 60-69, doi:10.1111/j.1399-0004.2009.01272.x (2010).
53. Anagnostopoulos, T. et al. G1738R is a BRCA1 founder mutation in Greek breast/ovarian cancer patients: evaluation of its pathogenicity and inferences on its genealogical history. Breast Cancer Res Treat 110, 377-385, doi:10.1007/s10549-007-9729-y (2008).
54. Zeegers, M. P., van Poppel, F., Vlietinck, R., Spruijt, L. & Ostrer, H. Founder mutations among the Dutch. Eur J Hum Genet 12, 591-600, doi:10.1038/sj.ejhg.5201151 (2004).
55. Mefford, H. C. et al. Evidence for a BRCA1 founder mutation in families of West African ancestry. Am J Hum Genet 65, 575-578, doi:10.1086/302511 (1999).
56. Sarantaus, L. et al. Multiple founder effects and geographical clustering of BRCA1 and BRCA2 families in Finland. Eur J Hum Genet 8, 757-763, doi:10.1038/sj.ejhg.5200529 (2000).
57. Campos, B. et al. Haplotype analysis of the BRCA2 9254delATCAT recurrent mutation in breast/ovarian cancer families from Spain. Hum Mutat 21, 452, doi:10.1002/humu.9133 (2003).
58. Papi, L. et al. Founder mutations account for the majority of BRCA1-attributable hereditary breast/ovarian cancer cases in a population from Tuscany, Central Italy. Breast Cancer Res Treat 117, 497-504, doi:10.1007/s10549-008-0190-3 (2009).
59. Infante, M. et al. Two founder BRCA2 mutations predispose to breast cancer in young women. Breast Cancer Res Treat 122, 567-571, doi:10.1007/s10549-009-0661-1 (2010).
60. Neuhausen, S. L. et al. Haplotype and phenotype analysis of nine recurrent BRCA2 mutations in 111 families: results of an international study. Am J Hum Genet 62, 1381-1388, doi:10.1086/301885 (1998).
61. Thorlacius, S. et al. A single BRCA2 mutation in male and female breast cancer families from Iceland with varied cancer phenotypes. Nat Genet 13, 117-119, doi:10.1038/ng0596-117 (1996).
62. Sidoni, T. et al. Identification and Characterization of BRCA1 and BRCA2 Founder Mutations. Current Women`s Health Reviews 8, 17-22, doi:http://dx.doi.org/10.2174/157340412799079192 (2012).
